# Prevalence of Chronic Obstructive Pulmonary Disease in Patients with Nontuberculous Mycobacterial Pulmonary Disease: A Systemic Review and Meta-Analysis

**DOI:** 10.3390/jpm14111089

**Published:** 2024-11-04

**Authors:** Hyun Lee, Jong Geol Jang, Youlim Kim, Kyung Hoon Min, June Hong Ahn, Kwang Ha Yoo, Min Gu Kang, Jong Seung Kim, Ji-Yong Moon

**Affiliations:** 1 Division of Pulmonary Medicine and Allergy, Department of Internal Medicine, Hanyang University College of Medicine, Seoul 04763, Republic of Korea; namuhanayeyo@hanyang.ac.kr; 2 Division of Pulmonology and Allergy, Department of Internal Medicine, Yeungnam University Medical Center, Yeungnam University College of Medicine, Daegu 42415, Republic of Korea; jang83@ynu.ac.kr (J.G.J.); fireajh@yu.ac.kr (J.H.A.); 3 Division of Pulmonary and Allergy, Department of Internal Medicine, Konkuk University Medical Center, Konkuk University School of Medicine, Seoul 05030, Republic of Korea; 20210617@kuh.ac.kr (Y.K.); 20010025@kuh.ac.kr (K.H.Y.); 4 Division of Pulmonary, Allergy and Critical Care Medicine, Department of Internal Medicine, Korea University Guro Hospital, Korea University College of Medicine, Seoul 08308, Republic of Korea; minkyunghoon@korea.ac.kr; 5 Research Institute of Clinical Medicine of Jeonbuk National University, Biomedical Research Institute of Jeonbuk National University Hospital, Jeonju 54907, Republic of Korea; kangmg@jbnu.ac.kr; 6 Department of Medical Informatics, Jeonbuk National University Medical School, Jeonju 54907, Republic of Korea; 7 Department of Otorhinolaryngology-Head and Neck Surgery, Jeonbuk National University Medical School, Jeonju 54907, Republic of Korea

**Keywords:** chronic obstructive pulmonary disease, systematic review, meta-analysis, nontuberculous mycobacterial pulmonary disease, prevalence

## Abstract

Background/Objectives: Nontuberculous mycobacterial pulmonary disease (NTM-PD) is an important comorbidity of COPD. Although many studies have reported an association between COPD and NTM-PD, no clear estimate of the prevalence of COPD and its effects on survival times in patients with NTM-PD is available. This study aimed to investigate the prevalence of COPD and its impact on survival in patients with NTM-PD. Methods: All studies reporting the prevalence of COPD in patients with NTM between 1952 and 2021 were searched using PubMed in May 2023. The inclusion criteria were studies about patients with NTM and COPD. A random-effects meta-analysis was performed in accordance with the Preferred Reporting Items for Systematic Reviews and Meta-Analyses guidelines. Results: The pooled overall prevalence of COPD in patients with NTM-PD was 28% (95% confidence interval [CI], 22–35). Patients with NTM-PD were six times more likely to have COPD than those without NTM-PD (pooled odds ratio [OR], 6.26; 95% CI, 3.37–11.65). Male patients with NTM-PD had a four-fold higher risk of COPD than females (OR, 3.81; 95% CI, 1.18–12.35). The co-existence of COPD and NTM-PD was significantly associated with an increased risk of mortality compared with NTM-PD without COPD (OR, 3.65; 95% CI, 1.28–10.40). Conclusions: COPD is common in patients with NTM-PD, and patients with NTM-PD had a six-fold increase in the odds of having COPD than those without NTM-PD. The presence of COPD and NTM-PD had a significant negative effect on survival. These findings may support the need to assess the presence of COPD in patients with NTM-PD and the potential negative effects associated with the co-existence of COPD and NTM-PD.

## 1. Introduction

Chronic obstructive pulmonary disease (COPD) is a chronic lung disease with increasing incidence that causes significant morbidity and mortality [1]. Although tobacco smoking is the major risk factor for COPD [2], non-smoking risk factors such as indoor and outdoor air pollution, occupational exposure, old age, low socioeconomic status, asthma, and infections also contribute to the development of COPD [3,4,5]. The Global Initiative for Chronic Obstructive Lung Disease (GOLD) recently revised its guidelines and proposed a taxonomy for COPD that emphasizes the importance of non-smoking risk factors for COPD. The GOLD committee introduced the term `infection-related COPD’, which includes conditions such as tuberculosis, human immunodeficiency virus, and recurrent childhood infections. This classification, referred to as COPD-I (COPD caused by infections), highlights the significant role of infections in the development of COPD. The GOLD committee also suggested that other causative infections contributing to COPD development should be further identified [1].

Nontuberculous mycobacterial pulmonary disease (NTM-PD) is a chronic and progressive infectious lung disease caused by various NTM species. The prevalence of NTM-PD is increasing worldwide and is associated with economic burden [6,7]. NTM-PD is commonly associated with structural lung disease, and COPD is a major comorbidity of NTM-PD [8,9]. Although many studies have previously reported an association between NTM-PD and COPD [10], the reported prevalence of COPD in patients with NTM has varied widely between studies [11,12,13,14,15,16,17]. Different study designs, regional differences, and small sample sizes might have contributed to the heterogeneity among studies. For example, studies conducted in South Korea and Japan have often reported higher prevalence rates of NTM-PD compared to studies from North America or Europe. These differences, whether related to environmental factors, genetic predispositions, or healthcare systems, make it challenging to accurately estimate the global prevalence of COPD in patients with NTM-PD [6,7,18].

The co-existence of other pulmonary comorbidities is known to be associated with poor treatment outcomes in NTM-PD [19]. For example, the prognosis of NTM-PD is worse when chronic pulmonary aspergillosis or COPD is present [19,20]. However, the estimated effects of co-existing COPD on NTM-PD have not been comprehensively elucidated, so a systematic review and meta-analysis on this subject is needed.

The aims of this systematic review and meta-analysis were (1) to comprehensively assess the prevalence of COPD in patients with NTM-PD by comparison with patients without NTM-PD, (2) to compare the prevalence of COPD in patients with NTM-PD with its prevalence in patients with pulmonary tuberculosis, and (3) to determine the effects of COPD on the survival of patients with NTM-PD.

## 2. Materials and Methods

### 2.1. Search Strategy

Three researchers (JSK, HL, JYM) independently searched all the relevant literature on PubMed up to May 2023, following the PRISMA (Preferred Reporting Items for Systematic Reviews and Meta-Analyses) guidelines. This study has not been registered in PROSPERO or any other databases. The search terms used were “nontuberculosis mycobacterium” and “chronic obstructive pulmonary disease”. The search criteria were as follows: (“ntm”[Journal] OR “ntm”[All Fields] OR “nontuberculous mycobacteri*”[All Fields] OR “mycobacteri*”[All Fields]) AND (“COPD”[All Fields] OR “chronic obstructive pulmonary disease”[All Fields]).

### 2.2. Selection of Literature

The inclusion criteria were as follows: (1) should contain information about NTM-PD; (2) should include content about COPD; (3) should have a prospective or retrospective study design; (4) should have been conducted with a human population; and (5) should be written in English.

The exclusion criteria were as follows: (1) papers in languages other than English; (2) no data about COPD; (3) no data about NTM; (4) case reports; (5) review articles; (6) studies without relevant data; and (7) NTM isolate

### 2.3. Data Extraction

Data extraction involved collecting information such as publication years, author names, NTM species, study design, number of COPD subjects, number of NTM-PD subjects, and years of follow up. The primary outcome was COPD incidence or prevalence, which we extracted when no control group for NTM was present and when a control group was present. We obtained dichotomous data for the following comparisons: the proportion of subjects with COPD among NTM-PD subjects compared with a control group, the proportion of subjects with COPD within the NTM group compared by sex, the ratio of subjects with COPD in NTM and tuberculosis (TB) groups, the proportion of subjects with COPD in groups with *Mycobacterium avium* complex (MAC) and *M. abscessus* complex (MAB), and the survival times of NTM-PD subjects with and without COPD.

### 2.4. Quality Assessment and Publication Bias

Three reviewers (Hyun Lee, Ji-Yong MOON, and Jong Seung Kim) graded the included studies based on their selection, comparability, exposure, and endpoint according to the Newcastle-Ottawa Scale guidelines.

### 2.5. Statistical Analysis

R software version 4.3.1 (R Foundation for Statistical Computing, Vienna, Austria) was used to conduct the meta-analysis. To test the proportion of subjects with COPD among NTM subjects without a control group, we used a single proportion test. For the other dichotomous data (the proportion of subjects with COPD among NTM subjects compared with a control group, the proportion of subjects with COPD within the NTM group by sex, the ratio of COPD subjects in NTM and TB groups, the proportion of subjects with COPD in groups with MAC and MAB, and the comparison of survival between NTM subjects with and without COPD), the odds ratio (OR) is presented as the effect size. Because each study was situated in distinct circumstances, we accounted for factors such as study location and ethnicity using a random effects model to present the effect size results. Hedge’s g and standard error were used for each primary outcome. Heterogeneity was assessed using I^2^ values, which range from 0% to 100% and represent the proportion of between-study variance to the total variance (combined between-study and within-study variance). An I^2^ value below 30% indicates low heterogeneity, and an I^2^ value of 70% or higher indicates substantial heterogeneity [21,22]. Egger’s test and funnel plots were used to detect potential publication bias [21,22]. The Duval and Tweedie trim-and-fill method, designed to address how missing studies can cause publication bias, was used to examine whether the overall effect size was likely to be changed by such bias.

## 3. Results

### 3.1. Characteristics of Included Studies

**Figure 1:**
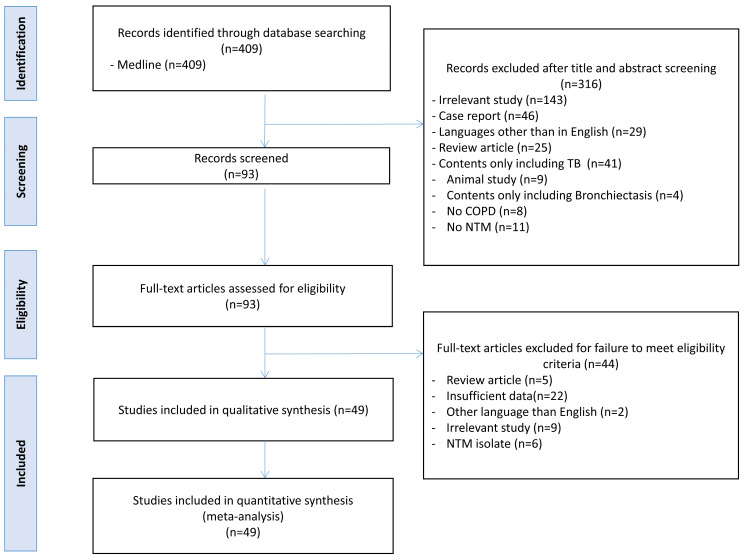
PRISMA 2020 flow diagram of studies included in the systematic review and meta-analysis. Abbreviations: COPD, chronic obstructive pulmonary disease; NTM, nontuberculous mycobacterial pulmonary disease.

A total of 409 studies were retrieved through the search query in May 2023. Of these, 316 studies met the exclusion criteria and were excluded after reviewing their titles and abstracts. Of the remaining 93 studies, 44 studies that did not meet the eligibility criteria were also excluded, leaving 49 studies for inclusion in the final meta-analysis and review, as shown in Figure 1. The characteristics of the 49 studies are summarized in Table 1 [10,11,12,13,14,15,16,17,23,24,25,26,27,28,29,30,31,32,33,34,35,36,37,38,39,40,41,42,43,44,45,46,47,48,49,50,51,52,53,54,55,56,57,58,59,60,61,62,63]. All included studies reported the incidence (1 study) or prevalence (48 studies) of COPD in subjects with NTM-PD, and all but 1 of them were cross-sectional in design (1 longitudinal study) [15].

### 3.2. Prevalence of COPD in Subjects with NTM-PD

**Figure 2:**
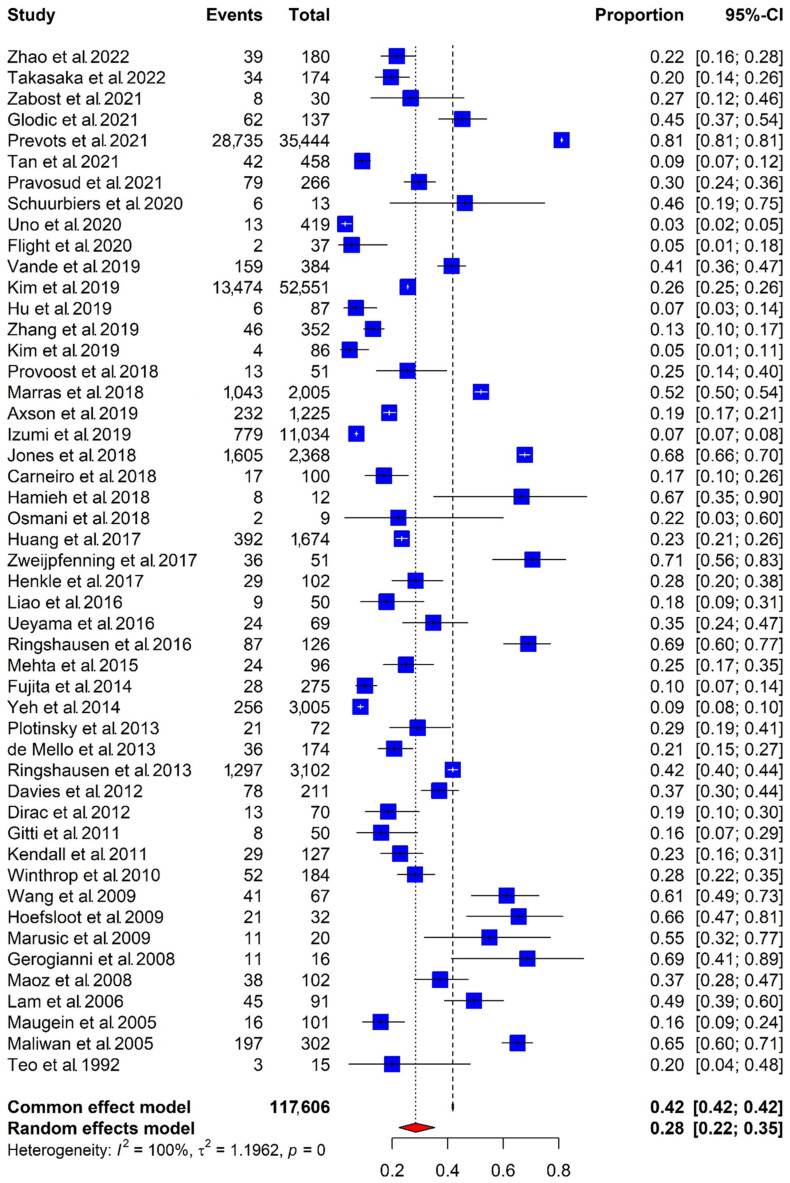
Pooled prevalence of COPD in subjects with NTM-PD [10,11,12,13,14,15,16,17,23,24,25,26,27,28,29,30,31,32,33,34,35,36,37,38,39,40,41,42,43,44,45,46,47,48,49,50,51,52,53,54,55,56,57,58,59,60,61,62,63]. Abbreviations: COPD, chronic obstructive pulmonary disease; NTM-PD, nontuberculous mycobacterial pulmonary disease; CI, confidential interval.

The overall pooled prevalence of COPD in subjects with NTM-PD was 28% (95% confidence interval [CI], 22–35): among the total of 117,606 NTM-PD subjects, 49,210 also had COPD. There was significant heterogeneity among the included studies (I^2^ = 100%, *p* < 0.01; Figure 2) that reported the prevalence of COPD in subjects with NTM-PD.

### 3.3. Association Between COPD and NTM-PD

**Figure 3:**
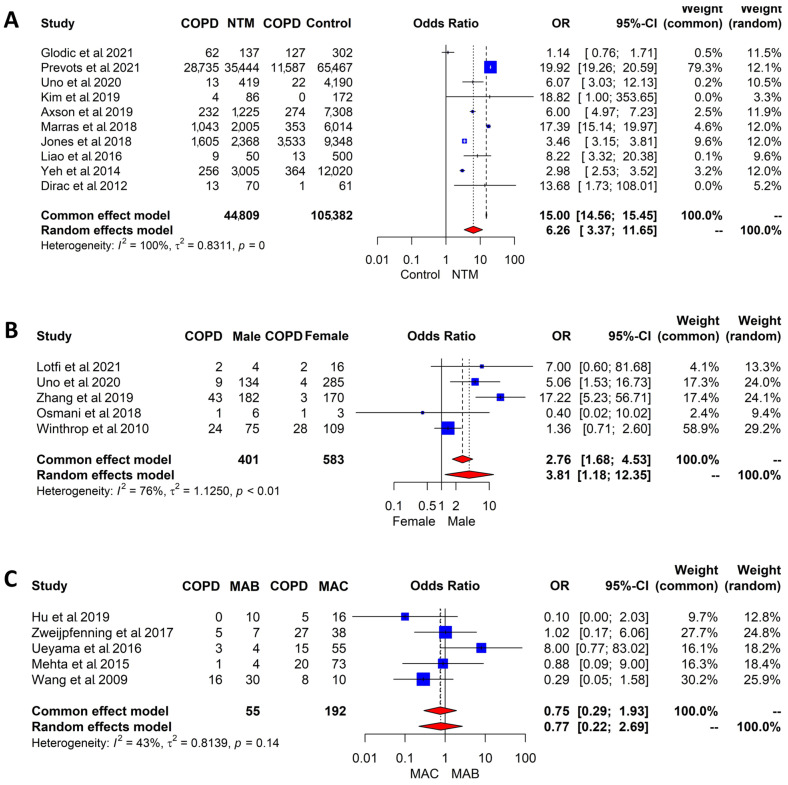
Forest plot of the (**A**) odds ratios of the prevalence of COPD in subjects with NTM-PD and a control group [10,11,13,14,15,17,25,36,37,51], (**B**) odds ratios of the prevalence of COPD in male versus female subjects with NTM-PD [17,27,34,40,54], and (**C**) odds ratio of COPD in MAC versus MAB in subjects with NTM-PD [33,42,44,46,55]. Abbreviations: COPD, chronic obstructive pulmonary disease; NTM-PD, nontuberculous mycobacterial pulmonary disease; CI, confidential interval; MAC, mycobacterium avium complex; MAB, mycobacterium abscessus; OR, odds ratio.

Ten studies examined the association between COPD and NTM-PD. Subjects with NTM-PD were six times more likely than those without NTM-PD to have COPD, with significant heterogeneity among studies (pooled OR, 6.26; 95% CI, 3.37–11.65; I^2^ = 100%, *p* = 0; Figure 3A).

### 3.4. Sex

Five studies reported the prevalence of COPD by sex in subjects with NTM-PD. When we combined the results of those five studies using a random effects model, male NTM-PD subjects had a four-fold higher risk of COPD than female NTM-PD subjects (OR, 3.81; 95% CI, 1.18–12.35), with substantial heterogeneity among studies (I^2^ = 76%; Figure 3B).

### 3.5. MAC-PD and MAB-PD

Five studies reported the prevalence of COPD in MAC-PD and MAB-PD. A random effects model across those five papers found no statistically significant difference in COPD prevalence between MAC-PD and MAB-PD (OR, 0.77; 95% CI, 0.22–2.69; Figure 3C). These results showed moderate heterogeneity (I^2^ = 43%).

### 3.6. NTM-PD and TB

**Figure 4:**
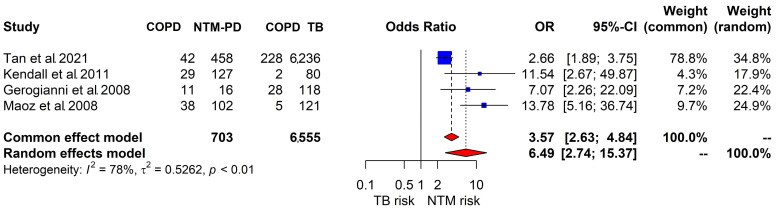
Forest plot comparing the risk of COPD in NTM-PD versus pulmonary TB [26,53,58,59]. Abbreviations: COPD, chronic obstructive pulmonary disease; NTM-PD, nontuberculous mycobacterial pulmonary disease; TB, tuberculosis; OR, odds ratio.

Four studies reported the COPD prevalence in subjects with pulmonary TB and NTM-PD. In the random effects model used for comparison, patients with NTM-PD had a higher risk of COPD than subjects with TB (OR, 6.49; 95% CI, 2.74–15.37; Figure 4). These results showed substantial heterogeneity (I^2^ = 78%).

### 3.7. Association Between COPD and Survival in Patients with NTM-PD

**Figure 5:**
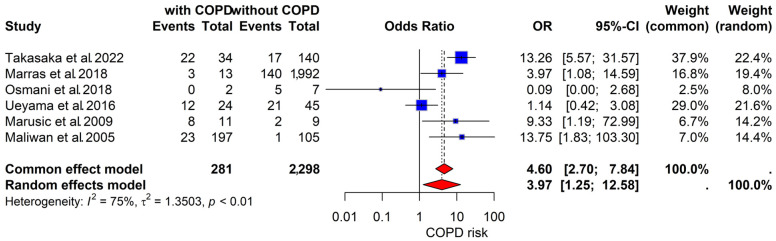
Effect of COPD on mortality during follow-up in subjects with NTM-PD [24,36,40,44,57,61]. Abbreviations: COPD, chronic obstructive pulmonary disease; NTM-PD, nontuberculous mycobacterial pulmonary disease; TB, tuberculosis; OR, odds ratio.

Six studies compared the survival of subjects with NTM-PD with and without COPD. The co-existence of COPD was significantly associated with an increased risk of mortality (OR, 3.97; 95% CI, 1.25–12.58; Figure 5). These results exhibited substantial heterogeneity (I^2^ = 75%, *p* < 0.01).

### 3.8. Publication Bias

In the funnel plot depicting the effects of the primary outcomes, most of the studies showed little evidence of publication bias (Supplementary Figure S1A,C,E,G,I). Egger’s regression test was conducted for COPD prevalence, one of the five primary outcomes, and yielded a *p*-value of 0.39, indicating no significant publication bias. We used Duval and Tweedie’s trim-and-fill method to account for potentially unpublished studies and found no significant change in the pooled odds ratio values (Supplementary Figure S1B,D,F,H,J).

## 4. Discussion

This study is the first comprehensive systematic review and meta-analysis to investigate the prevalence of COPD and its effects on the survival times of subjects with NTM-PD. We evaluated 49 studies with 117,606 subjects. The estimated pooled prevalence of COPD in subjects with NTM-PD was 28%, and subjects with NTM-PD were six times more likely to have COPD than those without NTM-PD. Additionally, the coexistence of NTM-PD and COPD was significantly associated with a 3.65-fold increased risk of mortality, highlighting the need for integrated management strategies.

This study shows that about 28% of subjects with NTM-PD also have COPD. Considering that the pooled prevalence of COPD in patients with a previous history of tuberculosis was 21%, the disease burden related to COPD in patients with NTM-PD seems to have been substantially under-recognized and neglected by clinicians. Although the study results show heterogeneity, indicating that the burden of COPD in patients with NTM-PD might vary widely by region and ethnicity, these results, nonetheless, highly suggest that more attention should be paid to the early detection and proper management of COPD in patients with NTM-PD. However, this heterogeneity also highlights the necessity of tailoring clinical approaches to specific populations, since varying environmental exposures, genetic predispositions, and healthcare infrastructures contribute to these regional differences. Understanding the high prevalence of COPD in NTM-PD patients has significant implications for both clinical practice and healthcare policy. Unfortunately, neither the current guidelines for NTM-PD diagnosis and treatment nor those for COPD pay attention to the comorbidity of these two diseases [64,65,66]. Integrating routine COPD screening into the management of NTM-PD patients could allow for earlier diagnosis and intervention, improving outcomes. For example, regular pulmonary function testing as a cost-effective strategy could help identify COPD in NTM-PD patients earlier, allowing for timely management.

The exact mechanism for the high prevalence of COPD in patients with NTM-PD remains unclear. One possible explanation is a bidirectional association between COPD and NTM-PD. NTM infection causes chronic airway inflammation, reduced mucociliary clearance, increased mucus secretion, and destruction of the lung, which are important mechanisms for the development of COPD [67,68]. COPD can also affect the development of NTM-PD. The destruction of the lung parenchyma in COPD reduces mucociliary clearance and damages the epithelium, which could reduce the clearance of inhaled pathogens and increase susceptibility to mycobacterial infection [69]. Other possible explanations are that NTM-PD and COPD share similar risk factors, including old age and low BMI [15,26,51].

In the past, the prevalence of COPD was higher in males than in females because males were more likely to smoke. More recently, however, no evidence of a sex difference in the prevalence of COPD has been found [64]. Because the prevalence of NTM-PD was higher in females than in males and COPD does not differ by sex, our finding of a stronger association between COPD and NTM-PD in males than females is interesting. Among subjects with NTM-PD, the prevalence of COPD was 3.81 times higher in males than females. The reasons for this finding remain unclear. One possible explanation is the inclusion of older studies in our analysis, which likely had a higher proportion of male smokers. Smoking has traditionally been more prevalent among men, especially in older generations, which may have contributed to the higher observed prevalence of COPD in males. Understanding the risk factors for a disease is important when designing a cost-effective strategy for its early detection, treatment, and prevention. Based on our findings, screening male patients with NTM-PD for COPD might be important. However, our study was observational, and we did not adjust for factors associated with the development of COPD, such as smoking status and age, so further studies are needed to clarify these associations.

The development, progression, and outcomes of NTM-PD vary by mycobacterial species, with the most clinically important species being MAC, *M. kansasii*, and MAB [70]. However, no previous studies have elucidated an association between COPD and mycobacterial species. We are the first to compare the prevalence of COPD between MAC-PD and MAB-PD, and we found no difference in the prevalence of COPD between them, suggesting that the development of airflow obstruction does not differ by NTM species but shares a common pathogenesis caused by NTM-PD. However, we analyzed only five studies with 250 people, so more studies on this issue are needed.

Generally, it is well known that having NTM-PD shortens patient lifespans. A previous study reported a hazards ratio of 1.47 in patients with NTM isolation or NTM-PD versus those without NTM isolation or NTM-PD [71]. In a Korean study, patients with NTM-PD had a two-fold increased risk of mortality compared with those without NTM-PD [72]. COPD is also associated with high mortality and is the third leading cause of death worldwide [64,73], so it was expected to further shorten the lifespans of patients with NTM-PD. In this study, the coexistence of COPD and NTM-PD was associated with 3.65-fold increased odds of mortality during follow-up compared with NTM-PD without COPD, indicating the importance of proper management for both diseases in patients with both conditions. Future studies of this finding and its implications are needed.

Our study has several limitations. First, most of the included studies were cross-sectional, so our results cannot explain casual interactions or temporal relationships between COPD and NTM-PD. Second, the included studies have significant heterogeneity in their study designs, populations, and geographic locations, suggesting that the estimated prevalence should be interpreted with caution. Third, we included only published studies in this systematic review and meta-analysis, which could cause publication bias, but we performed statistical tests and found no significant publication bias. Lastly, the number of studies included in some parts of the analysis was relatively small, particularly for those comparing the prevalence of COPD in patients with NTM-PD and those with TB. Despite an extensive literature search, there was a notable lack of studies specifically addressing COPD prevalence in TB patients, limiting our ability to conduct a comprehensive analysis of this comparison. This restricts the strength of our findings in this area, and future research is needed to address this gap by focusing on both NTM-PD and TB populations.

Despite these limitations, this is the first systematic review and meta-analysis to evaluate the prevalence of COPD in patients with NTM-PD, and our comprehensive literature search returned 49 studies with 117,606 subjects.

## 5. Conclusions

COPD is common in patients with NTM-PD, and patients with NTM-PD have a two-fold increase in their odds of having COPD. The presence of COPD has a significant negative effect on the survival time of patients with NTM-PD. Future studies could focus on elucidating the mechanisms that link COPD and NTM-PD. Additionally, longitudinal studies are needed to investigate the temporal associations between these diseases to clarify their bidirectional relationship, to assess the long-term impact of COPD on the life span of patients with NTM-PD, and to identify risk factors for COPD in patients with NTM-PD.

## Figures and Tables

**Table 1:** Characteristics of the 49 studies included in this meta-analysis.

Author and Year	Country	Study Design	Quality Score	Size of Study Population		Follow-Up (Years)
				COPD	NTM-PD (Control)	
Zhao 2022 [23]	China	Retrospective CS	7	39	180	2018–2021
Takasaka 2022 [24]	Japan	Retrospective CS	7	34	174	2014–2018
Zabost 2021 [12]	Poland	Retrospective CS	8	3	16	2011–2020
Glodić 2021 [25]	Croatia	Retrospective Cohort	8	62	137 (302)	2006–2015
Prevots 2021 [11]	USA	Retrospective Cohort	6	28,735	35,444 (65,467)	2007–2015
Tan 2021 [26]	China	Prospective CS	7	42	458	2019–2020
Lotfi 2021 [27]	Iran	Retrospective CS	6	4	20	2018–2019
Pravosud 2021 [28]	USA	Retrospective CS	5	79	266	2016–2017
Schuurbiers 2020 [29]	Netherlands	Retrospective CS	5	6	13	2010–2018
Uno 2020 [17]	Japan	Retrospective CS	6	13	419 (4190)	2014
Flight 2020 [30]	UK	Retrospective CS	7	2	37	2014–2019
Vande 2019 [31]	Belgium	Retrospective CS	7	159	384	2010–2017
Kim 2019 [32]	Korea	Retrospective CS	6	13,474	52,551	2009–2015
Hu 2019 [33]	China	Retrospective CS	8	6	87	2017–2018
Zhang 2019 [34]	Singapore	Retrospective CS	8	46	352	2012–2016
Kim 2019 [13]	Korea	Retrospective CS	7	4	86 (172)	2010–2014
Provoost 2018 [35]	France	Retrospective CS	7	13	51	2013–2016
Marras 2018 [36]	US	Retrospective CS	7	1043	2005 (6014)	2007–2016
Axson 2019 [37]	UK	Retrospective CS	6	232	1225 (7308)	2007–2015
Izumi 2019 [16]	Japan	Retrospective CS	7	550	3526	2009–2014
Jones 2018 [10]	US	Retrospective CS	6	1605	2368 (9348)	2008–2012
Carneiro 2018 [38]	Brazil	Retrospective CS	8	17	100	2003–2013
Hamieh 2018 [39]	Lebanon	Retrospective CS	6	8	12	2004–2016
Osmani 2018 [40]	USA	Retrospective CS	8	2	9	2007–2014
Huang2017 [41]	Taiwan	Retrospective CS	8	392	1674	2008–2014
Zweijpfenning 2017 [42]	Netherlands	Retrospective CS	7	36	51	2008–2013
Henkle 2017 [43]	USA	Retrospective CS	9	29	102	2005–2015
Ueyama 2016 [44]	Japan	Retrospective CS	7	24	69	1992–2013
Liao 2016 [14]	Taiwan	Retrospective CS	9	9	50 (500)	1992–2011
Ringshausen 2016 [45]	Germany	Retrospective CS	7	87	126	2009–2014
Mehta 2015 [46]	Canada	Retrospective CS	7	24	96	2003–2010
Fujita 2014 [47]	Japan	Retrospective CS	8	28	275	2001–2013
Yeh 2014 [15]	Taiwan	Retrospective cohort	7	256	3005 (12,020)	1996–2010
Plotinsky 2013 [48]	USA	Retrospective CS	7	21	72	1993–2006
de Mello 2013 [49]	Brazil	Retrospective CS	9	36	174	1993–2011
Davies 2013 [50]	UK	Retrospective CS	7	21	57	2000–2007
Dirac 2012 [51]	USA	Retrospective CS	8	13	70 (61)	2012
Gitti 2011 [52]	Greece	Retrospective CS	7	8	50	2000–2009
Kendall 2011 [53]	USA	Retrospective CS	7	29	127	2005–2006
Winthrop 2010 [54]	USA	Retrospective CS	8	52	184	2005–2006
Wang 2009 [55]	Taiwan	Retrospective CS	8	41	67	2004–2005
Hoefsloot 2009 [56]	Netherlands	Retrospective CS	9	21	32	2002–2006
Marusić 2009 [57]	Croatia	Retrospective CS	7	11	20	1980–2005
Gerogianni 2008 [58]	Greece	Retrospective CS	7	11	16	2004–2006
Maoz 2008 [59]	Israel	Retrospective CS	7	38	102	1999–2005
Lam 2006 [60]	USA	Randomized Controlled Trial	Unclear risk *	45	91	2000–2003
Maugein 2005 [61]	France	Retrospective CS	6	16	101	2000–2002
Maliwan 2005 [62]	USA	Retrospective CS	7	197	302	1952–1995
Teo 1992 [63]	Singapore	Retrospective CS	5	3	15	1976–1988

* Cochrane Risk Of Bias tool 3.2. Prevalence of COPD in subjects with NTM. Quality score was assessed using the Newcastle-Ottawa Scale (observational study) or Cochrane Risk Of Bias tool (randomized controlled trial). Abbreviations: COPD, chronic obstructive pulmonary disease; NTM-PD, nontuberculous mycobacterial pulmonary disease; CS, cross-sectional design.

## Data Availability

The datasets used and/or analyzed during the current study are available from the corresponding author upon reasonable request due to privacy and ethical restrictions.

## Supplementary Materials

The following supporting information can be downloaded at https://www.mdpi.com/article/10.3390/jpm14111089/s1: Figure S1: Funnel plot of the (A) proportion of subjects with COPD among NTM-PD subjects compared with a control group; (B) data from A after the application of Duval’s trim-and-fill method; (C) proportion of subjects with COPD in the NTM-PD group by sex; (D) data from C after the application of Duval’s trim-and-fill method; (E) comparison of COPD prevalence between NTM-PD and TB; (F) data from E after the application of Duval’s trim-and-fill method; (G) proportion of patients with COPD in subjects with MAC-PD and MAB-PD; (H) data from G after the application of Duval’s trim-and-fill method; (I) comparison of mortality between NTM-PD with and without COPD; (J) data from I after the application of Duval’s trim-and-fill method.
