# Supplementary material for: Prevalence of Chronic Obstructive Pulmonary Disease in Patients with Nontuberculous Mycobacterial Pulmonary Disease: A Systemic Review and Meta-Analysis
Source: J Pers Med. 2024 Nov 4;14(11):1089. doi: 10.3390/jpm14111089 (PMC11595912; doi:10.3390/jpm14111089)
Supplement: Supplementary file 1 [file jpm-14-01089-s001.zip › jpm-3253642-supplementary.pdf]

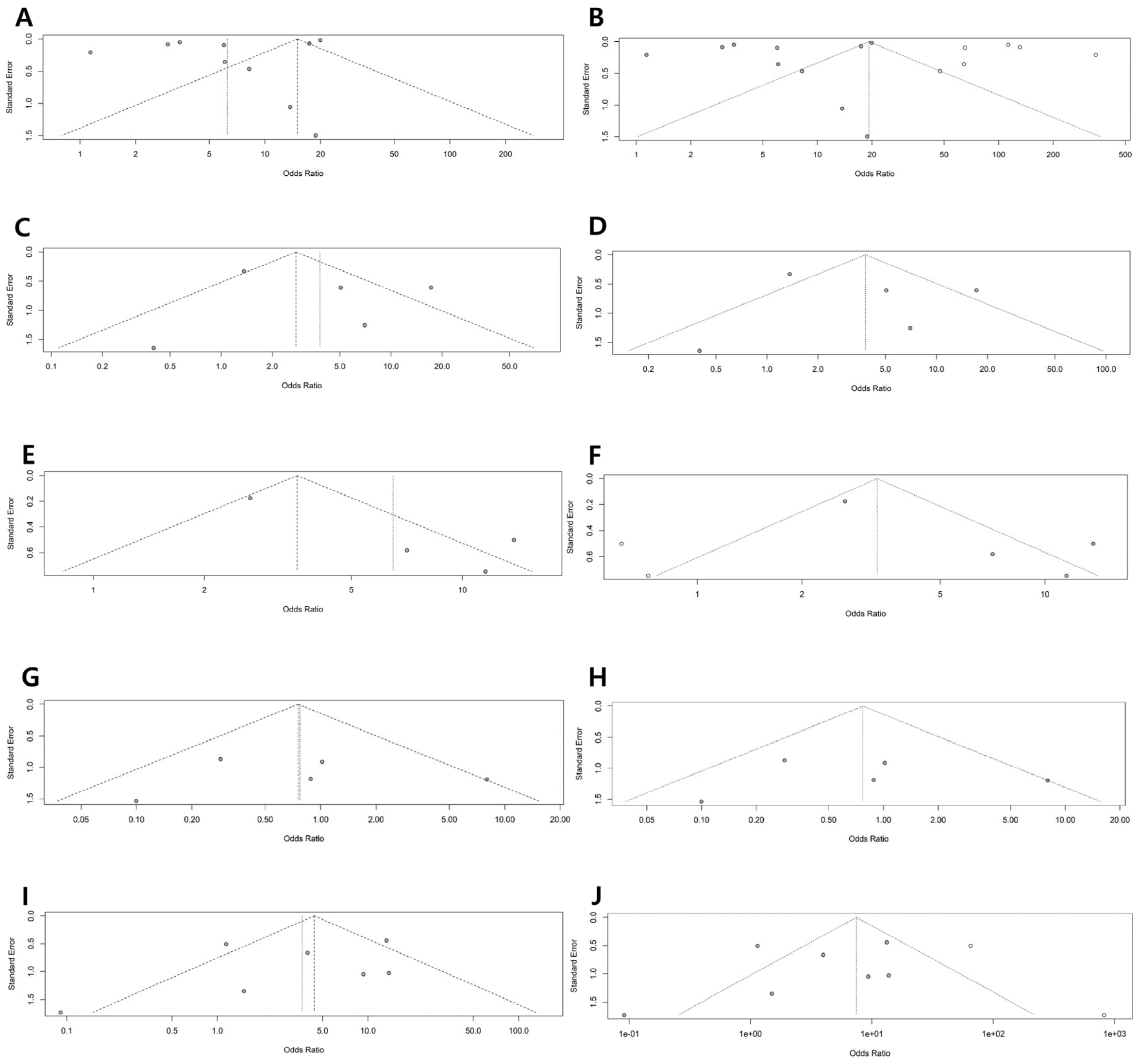

Figure S1. Funnel plot of the (**A**) proportion of subjects with COPD among NTM-PD subjects compared with a control group; (**B**) data from A after the application of Duval's trim-and-fill method; (**C**) proportion of subjects with COPD in the NTM-PD group by sex; (**D**) data from C after the application of Duval's trim-and-fill method; (**E**) comparison of COPD prevalence between NTM-PD and TB; (**F**) data from E after the application of Duval's trim-and-fill method; (**G**) proportion of patients with COPD in subjects with MAC-PD and MAB-PD; (**H**) data from G after the application of Duval's trim-and-fill method; (**I**) comparison of mortality between NTM-PD with and without COPD; (**J**) data from I after the application of Duval's trim-and-fill method.
